# Harmony: EEG/MEG Linear Inverse Source Reconstruction in the Anatomical Basis of Spherical Harmonics

**DOI:** 10.1371/journal.pone.0044439

**Published:** 2012-10-11

**Authors:** Yury Petrov

**Affiliations:** Psychology Department, Northeastern University, Boston, Massachusetts, United States of America; University of Cambridge, United Kingdom

## Abstract

EEG/MEG source localization based on a “distributed solution” is severely underdetermined, because the number of sources is much larger than the number of measurements. In particular, this makes the solution strongly affected by sensor noise. A new way to constrain the problem is presented. By using the anatomical basis of spherical harmonics (or spherical splines) instead of single dipoles the dimensionality of the inverse solution is greatly reduced without sacrificing the quality of the data fit. The smoothness of the resulting solution reduces the surface bias and scatter of the sources (incoherency) compared to the popular minimum-norm algorithms where single-dipole basis is used (MNE, depth-weighted MNE, dSPM, sLORETA, LORETA, IBF) and allows to efficiently reduce the effect of sensor noise. This approach, termed Harmony, performed well when applied to experimental data (two exemplars of early evoked potentials) and showed better localization precision and solution coherence than the other tested algorithms when applied to realistically simulated data.

## Introduction

The EEG (electro-encephalography) method is based on amplifying and recording weak electrical currents produced by an active brain. Compared to other brain-imaging methods EEG is truly non-invasive and inexpensive. EEG, along with its ‘magnetic’ cousin MEG, is the only non-invasive brain-imaging method that has high-enough temporal resolution to track the full dynamics of brain events. Because the method has a limited spatial resolution EEG was not considered to be an ‘imaging’ technique until recently. Skull has low conductivity compared to adjacent head tissues which strongly diffuses electrical currents generated by brain activity. The problem of reconstructing brain activity from its blurred image recorded by sensors positioned outside of the head is an example of the inverse problem termed “source localization”.

Source-localization includes the two steps: creating a head model which describes how the head volume conducts electrical current, and fitting the model into the recorded data. Since the introduction of the MRI technique anatomical aspects of head modeling have became more manageable (tissue conductivities still remain a topic of debate as discussed in chs. 4 and 6 of [1] and also in [2]–[5]), and many source-localization algorithms based on fairly realistic boundary element (BEM) and finite element (FEM) head models have been proposed ([6]–[8], for examples).

The localization results also strongly depend on what technique is used to fit the observed data. The focus of this study is on the ‘distributed’ or ‘nonparametric’ type of the inverse solution, where thousands of current dipoles (sources) at fixed locations are used to fit the data using the minimum-norm (L2) metric. The purpose was not to propose a new golden standard, but simply to show how choosing the source basis made of globally smooth functions improves source reconstruction within the L2 norm framework. Such choice can be considered as the first step in more complex source localization algorithms (see the Discussion). Conversely, a ‘parametric’ or equivalent source dipole (ECD) solution finds unknown locations for a small number of source dipoles. The main advantage of the ‘distributed’ L2 solution is that after fixing the dipole positions the forward problem becomes linear (defined in terms of the forward matrix 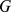), and can be easily solved by inversion. The drawback of this approach is that the inverse problem is hugely underdetermined. In physical terms, infinitely many different source combinations can produce the observed distribution of potential on the scalp. In mathematical terms, the number of independent signals spanning the signal space, which is less than the number of EEG/MEG sensors due to the sensor cross-talk [9] is much smaller than the number of the fitted dipole amplitudes spanning the source space (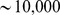). Hence only a small number of vectors in the source space equal to the number of uncorrelated signals are effectively chosen by the linear inverse to span the solution, which is strongly affected by this choice. The most direct choice of the solution subspace is to use the rows of 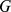, the approach termed “minimum norm” [10], [11]. This popular approach which in its most basic form reduces to the Moore-Penrose pseudoinverse produces the lowest total power of the source currents in the solution because it excludes any source combinations falling within the null-space of the forward matrix 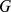. Many source localization algorithms (e.g., MNE [11], WMNE [11], [12], LORETA [13], sLORETA [14], dSPM [15], LAURA [16], FOCUSS [17]) are derivatives of this approach [8], [9], [18].

Here a new approach to EEG/MEG source localization, termed Harmony, that improves on some of the existing techniques is described. In addition to showing superior performance the proposed approach has a number of practical advantages: it easily interpolates the solution onto any cortical mesh and describes brain activity in the form of a spatial spectrum, analogous to the widely used temporal (Fourier) spectrum. The goal of this study was to show that source reconstruction in a small basis set comprising global smooth functions, such as spherical harmonics or spherical splines, significantly improves the source reconstruction quality as compared to the commonly used basis set of tens of thousands discrete cortical dipoles. To make it a fair comparison, algorithms with this commonly used basis set and a bare minimum of extra features (depth-weighting and spatial smoothing) were compared to Harmony: MNE (minimum-norm), WMNE (weighted minimum norm), IBF (Informed Basis Functions) [19], and LORETA. Effects of the solution normalization (dSPM, sLORETA) were also considered. Various additional manipulations, such as iteratively re-weighting the solution or choosing the solution's prior within the framework of empirical Bayes approaches [17], [20]–[24] were not considered here because from the practical point of view these manipulations are separate from choosing the basis set for source reconstruction and can be later used to augment Harmony as well as other algorithms.

The idea of using a small (sparse) basis set to fit EEG/MEG data was previously investigated in the contexts of both the ‘distributed’ and ‘parametric’ algorithms, where, eventually, a small set of source dipoles was sought. For the ‘distributed’ approach, iterative schemes where source dipole weights at a given iteration are chosen based on the results of the previous iteration were used to truncate the resulting number of active sources to a small number, e.g., the FOCUSS algorithm [17], [25]. For the ‘parametric’ approaches MUSIC and RAP-MUSIC algorithms [26], [27] were proposed to resolve the critical issue of choosing the ‘correct’ number of dipoles. Preprocessing of raw data by the Independent Component Analysis (ICA) was used to reduce the number of simultaneously fitted dipoles [28]. When applied to the localization of simulated [29] and real local sources [30] the method showed good results. To better the fit while staying within the framework of parametric algorithms source dipoles were sought to be replaced by source multipoles [31], [32] or local source patches [33]–[36]. These approaches essentially aim to fit not only the location but also local bulk properties of current sources such as their spatial extent and curvature. In a certain sense the approach proposed here applies the same ideas globally by treating each cortical hemisphere as a single extended source with known (cortical) curvature and extent and unknown distribution of activity across its surface.

## Results

**Figure 1:**
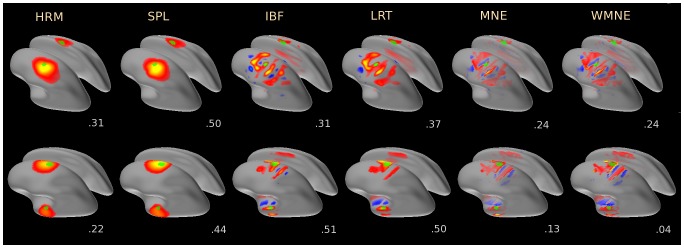
Representative reconstructions of two source configurations (different hemispheres - top row, the same hemisphere - bottom row) by the tested algorithms. The results are shown on the inflated cortices: the 37-dipole source patches are shown by the green patches, dipole orientations for the sources and the solutions were constrained to be orthogonal to the cortical surfaces in this case. Color indicates amplitude and direction of cortical currents: inward – cold, outward – hot. Numbers underneath each panel give AUC measure of the reconstruction quality as given by (23).

**Figure 2:**
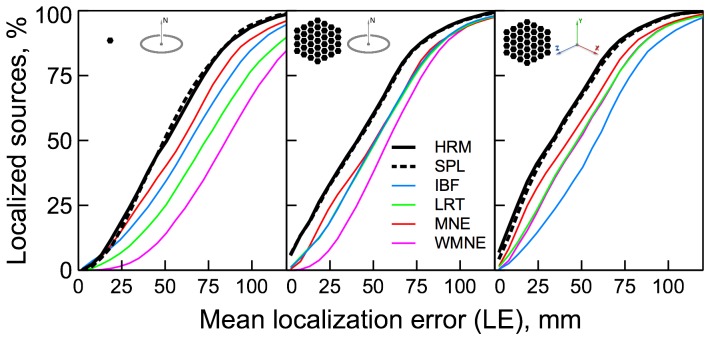
Localization errors for the tested algorithms. The localization error is plotted along the x-axis. The percentage of sources reconstructed with localization error smaller than a given x-value is plotted along the y-axis. Results for different source configurations (illustrated by the insets) are shown in the corresponding panels.

**Figure 3:**
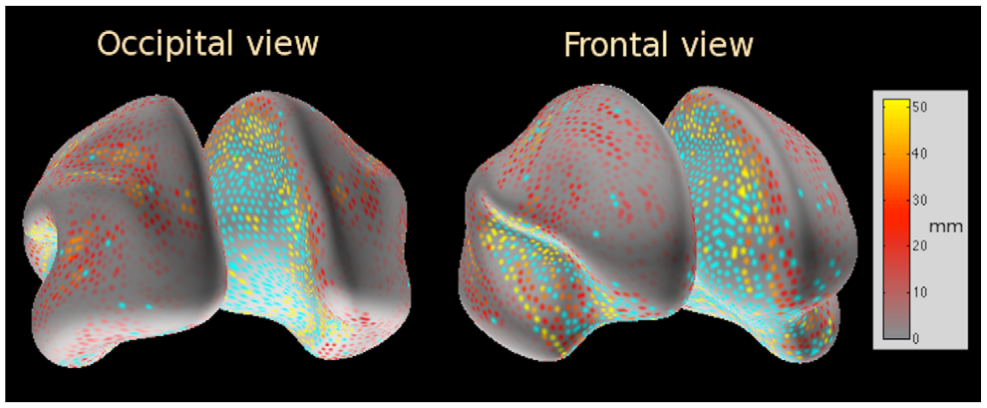
A map of Harmony localization errors based on the results of a single source simulation. Colored spots mark tested locations, spot colors indicate the localization error as given by the colorbar. Cyan colored spots (cyan is not represented on the color bar) mark sources which could not be localized significantly above chance level.

**Figure 4:**
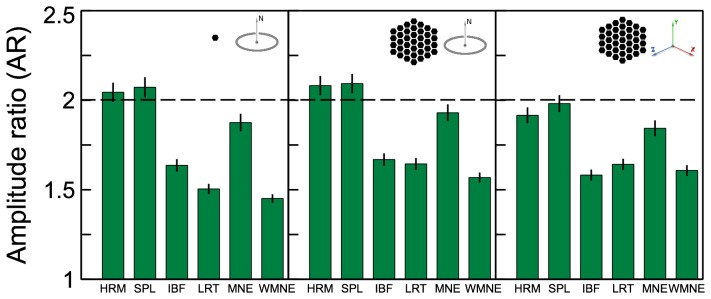
Amplitude ratio between the left and right reconstructed sources. The dashed line indicates the true amplitude ratio = 2 between the two simulated sources. Results for different source configurations (illustrated by the insets) are shown in the corresponding panels.

**Figure 5:**
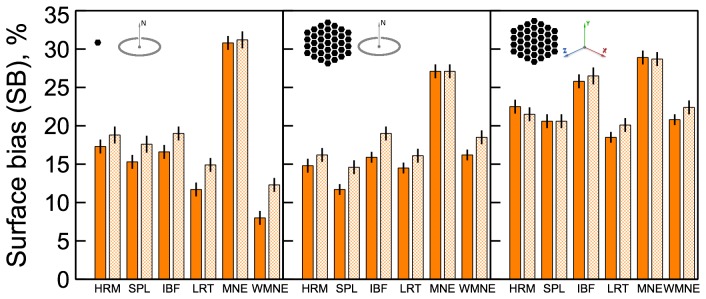
Surface bias (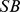) comparison among different algorithms. Results for different source configurations (illustrated by the insets) are shown in the corresponding panels. Solid bars show results for sources in different cortical hemispheres, hashed bars - results for both sources in the same hemisphere.

**Figure 6:**
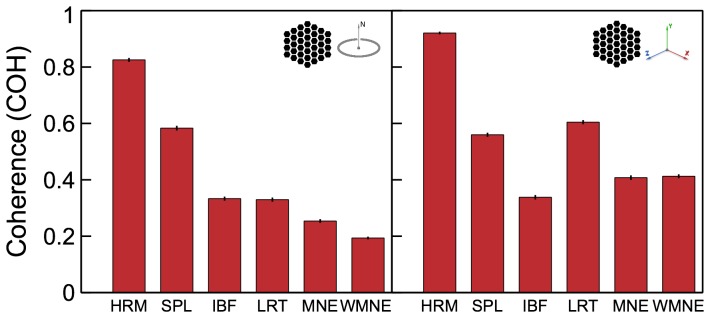
Coherence 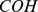 measure for the tested algorithms. Results for different source configurations (illustrated by the insets) are shown in the corresponding panels.

**Figure 7:**
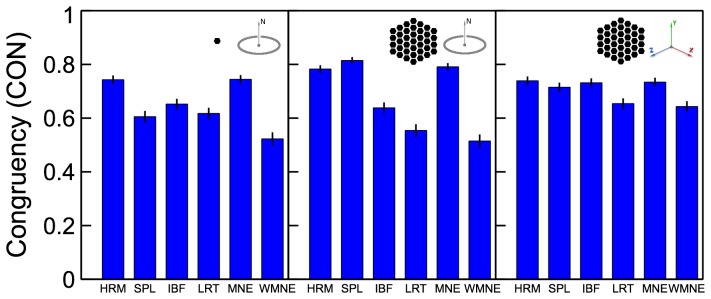
Congruency (width – error correlation) 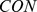 measure for the tested algorithms. Results for different source configurations (illustrated by the insets) are shown in the corresponding panels.

**Figure 8:**
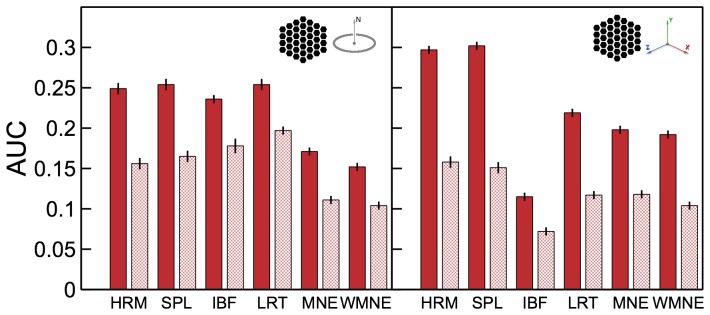
Area under the ROC curve 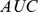 measure for the tested algorithms. Results for different source configurations (illustrated by the insets) are shown in the corresponding panels. Solid bars show results for sources in different cortical hemispheres, hashed bars - results for both sources in the same hemisphere.

**Figure 9:**
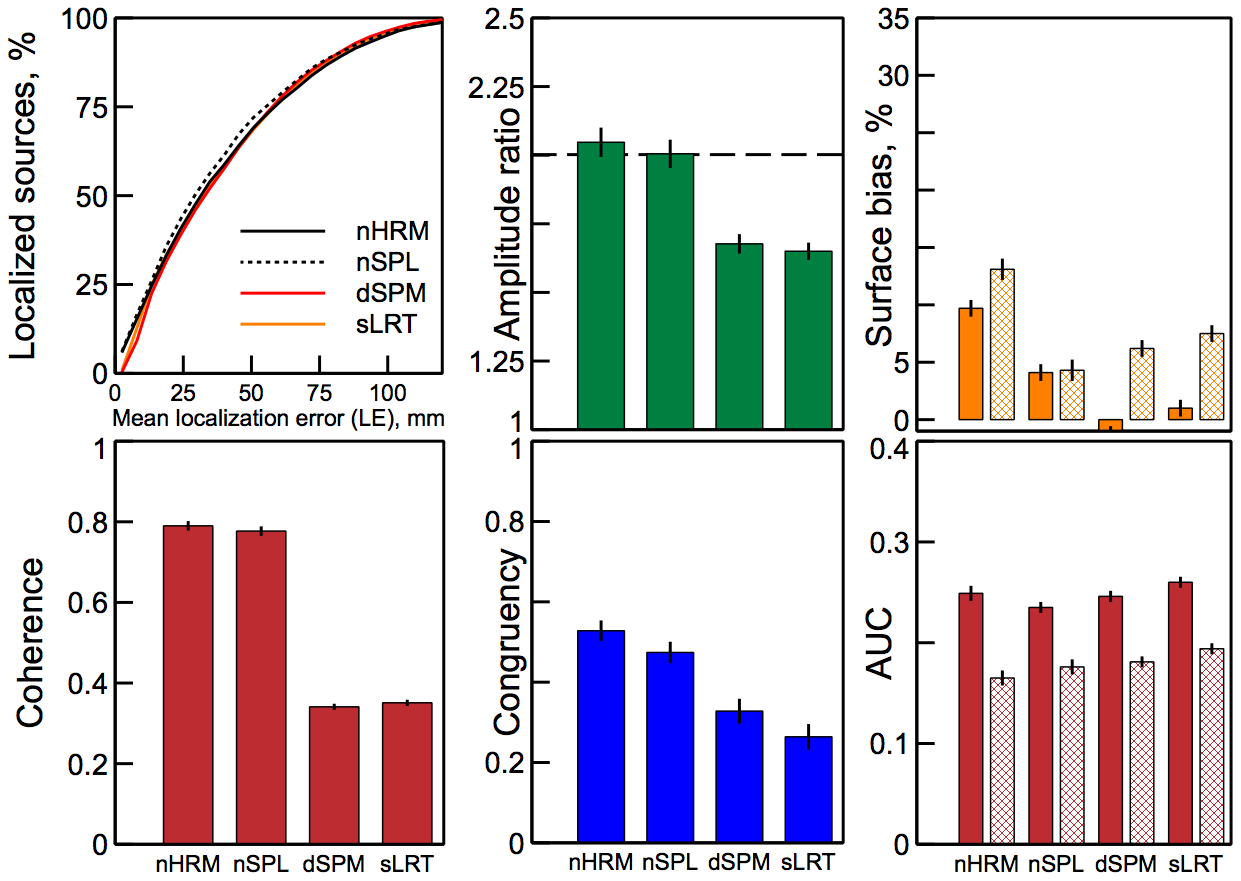
The six measures of the reconstruction quality presented in **Figures 2**–**8** applied to normalized solutions. Solid bars indicate results for sources on opposite hemispheres, hashed bars for sources on the same hemisphere. nHRM and nSPL denote Harmony solutions in the basis of spherical harmonics and spherical splines normalized by the standard deviation of the solution for each current dipole, i.e., dSPM-like.

**Figure 10:**
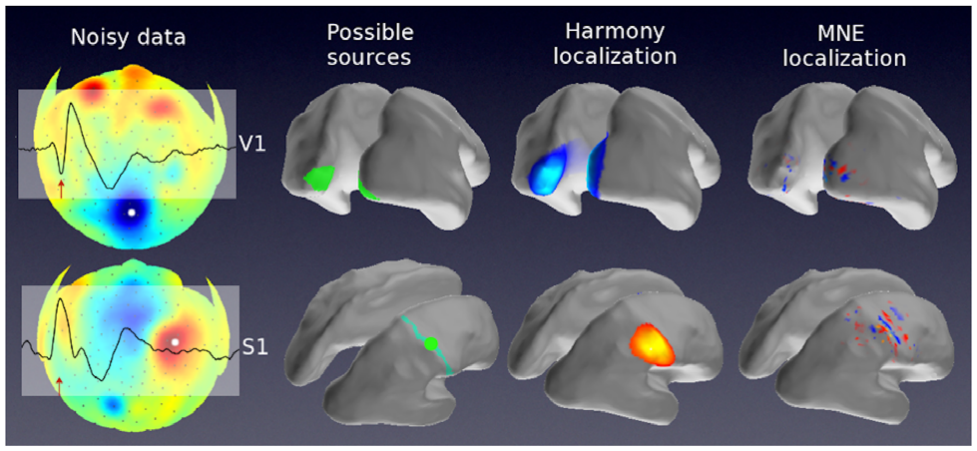
Localizations of the observed evoked potentials: visually evoked (top) and somatosensory evoked (bottom). From left to right: (1) A snapshot of the observed ERP potentials interpolated and shown on a flattened scalp. EEG sensor positions are marked with dark dots, the overlay shows ERP time course at the scalp location marked with a white dot. The red arrow indicates the time point at which the displayed scalp potentials were recorded (N1: 97 msec for visual stimulation, 86 msec for tactile stimulation). (2) The expected cortical activations: labels refer to the expected sites of cortical activation: V1 – primary visual cortex, and S1 – primary somatosensory cortex. (3) Harmony reconstruction results. (4) MNE reconstruction results.

Results of source reconstructions for simulated and real EEG data are presented in Figures 1–10. Error bars in all plots represent 95% confidence intervals for the plotted value. As described in detail in the Materials and Methods section the simulations used two types of sources: single-dipoles and extended 37-dipole patches, and two types of reconstructions: with and without dipoles being constrained to be orthogonal to the cortical surface. In either case the actual simulated sources were orthogonal to the cortex. Typically, three types of reconstructions are compared in each figure: single-dipole orientation-constrained, extended patch orientation-constrained, and extended patch unconstrained. Single-dipole unconstrained reconstructions did not show anything beyond what can be seen from the analysis of the remaining three cases and are not discussed. In addition, the two simulated source patches were located either in different cortical hemispheres or in the same hemisphere. Only surface bias (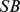) and area under the ROC curve (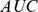) metrics could be applied in the latter case (see Materials and Methods for explanation), the corresponding results are shown with hashed bars in Figures 5 and 8.

### Qualitative comparison

Figure 1 illustrates representative source reconstructions produced by the tested algorithms for two source configuration. A configuration with sources positioned in opposite cortical hemispheres is shown in the top row, a configuration with sources in the same hemisphere – in the bottom row. In the top row the left-hemisphere source was twice the amplitude of the right-hemisphere source, in the bottom row the top source was twice the amplitude of the bottom source. The source patches are shown by the green shapes overlaid on the inflated cortices. In this and following figures HRM, SPL, IBF, LRT, MNE, WMNE stand, respectively, for Harmony in the basis of spherical harmonics, Harmony in the basis of spherical splines, Informed Basis Functions, LORETA, MNE, and weighted MNE algorithms. Normalized MNE reconstructions (dSPM and sLORETA) were not shown in Figure 1, they were similar to the MNE reconstructions with less surface bias and more spatial dispersion [37]. Orientation-constrained reconstructions are shown here because they allow to illustrate differences between the solutions in a simple manner by using colormap to represent both magnitude and direction of the current dipoles: cold colors for inward currents, hot colors for outward currents. The colormap was scaled optimally for each algorithm's result and hence the activity patterns are shown on various scales; Harmony reconstructions had the largest dipole amplitudes, MNE reconstructions - the weakest.

The two Harmony solutions (spherical harmonics, HRM, and spherical splines, SPL) clearly stand out. Harmony produced coherent smooth solutions located close to the sources and without an apparent surface bias. The spherical harmonics and spherical splines solutions look very similar. The splines solution appears to be slightly ‘tighter’ but also slightly less coherent: it shows some artefacts of the spline ‘mesh’, where the centers of several neighboring splines appear as ‘grains’ inside the left hemisphere hotspot.

The remaining algorithms produced visibly incoherent solutions characterized by stripes of activity aligned along gyri with alternating ‘hot’ and ‘cold’ colors corresponding to alternating outward and inward cortical currents. Note that these artefacts would not be apparent if the solutions were displayed on folded cortical surfaces using a colormap indicating only the absolute values of the current and not its direction, which is a common practice. The local alternations in the current's direction similarly exist in orientation-free reconstructions but are less apparent in this case because the dipole magnitudes may vary fairly smoothly even as their directions vary abruptly across cortical surface.

The stripy appearance is a well known tendency of distributed solutions [16], [38] which result from two factors, both related to the minimum-norm constraint: (i) the surface bias of the solution favoring gyri, and (ii) the sign reversal across sulci. The surface bias artifacts were prominent even though WMNE, LORETA, and IBF algorithms biased solution to deeper sources via the same depth-weighting prior (15). To explain why without a smoothness constraint (MNE, WMNE) or with insufficient smoothness constraint (LORETA, IBF) the solution shows the characteristic sign reversal across sulci one needs to consider that a minimum-norm (minimum power) solution precludes currents located nearby in space having opposite directions because in this case they mutually cancel. For sources inside a sulcus this means that their sign must switch abruptly (from inward to outward or vice versa) between the opposite banks of a sulcus in order for the currents not to oppose each other.

Next to each reconstruction the corresponding 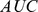 metric is shown, the metric is discussed in detail in the next section. It is worth noting that for the case of small source patches presented here the metric can vary significantly with only little visually apparent difference between the solutions (compare HRM and SPL reconstructions). Also, the metric does not penalize incoherent solutions (compare HRM and LRT reconstructions).

### Quantitative comparison

Six measures defined in the Materials and Methods section were used to compare the quality of source reconstructions among the tested algorithms. All six measures were used for configurations with sources in different hemispheres. Only the surface bias, 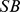, and area under the ROC curve, 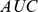, measures were used for configurations with sources in the same cortical hemisphere. The remaining measures could not be used without somehow defining which activated dipoles reconstruct what simulated source. When the sources were in different hemispheres only the dipoles belonging to the same hemisphere were used for each source. When the sources were in the same hemisphere no such obvious classification could be made.

#### Localization error, 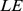


The localization error (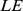) results are shown in Figure 2. The 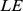 value given by (17) is plotted along the x-axis, and the percentage of sources localized with a smaller error than the given x-value is plotted along the y-axis. From left to right the three panels display results for the single-dipole orientation-constrained, 37-dipole orientation-constrained, and 37-dipole unconstrained reconstructions respectively. These three configurations are illustrated by the cartoons shown at the top of each panel.

Both the spherical harmonics and spherical splines Harmony algorithms demonstrated superior performance compared to the other tested algorithms. The advantage was particularly large for the extended patch configurations and for those sources which could be localized with better than 3 cm accuracy. This is important given that practical significance of reconstructions with larger localization errors is questionable.

Removing the orientation constraint somewhat decreased localization errors of all algorithms except IBF. The improvements were particularly significant for WMNE. Although the unconstrained solution had less localization error it was characterized by a significantly larger surface bias (Figure 5).

#### Localization error map

Figure 3 displays a map of localization errors for the Harmony algorithm (spherical harmonics and splines produced almost identical results). This map can be used as a rough estimate of the reliability of source localization over cerebral hemispheres. Unlike the rest of the simulations this map was calculated for a single cortical source. The source was the 37-dipole patch, its dipole strengths were set in the same way as for the two-source simulations, i.e., to produce 10–12 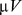 EEG potentials for superficial sources. The source was positioned at the nodes of a uniform grid (4th subdivision of icosahedron, 2,562 nodes) covering each hemisphere. Experimental noise was added the same way as for the two-source simulations. Orientation constraint was used for the solution. The localization error (LE) at the tested locations is indicated by colormap in Figure 3. ‘Hot’ locations mark large localization errors, ‘cooler’ locations mark smaller errors.

From the practical point of view it is useful to know where the reconstructions were not significantly better than chance. This statistical measure was computed by comparing localization results for the simulated signal (plus the added sensor noise) to localization results for noise-only data. For the latter case the simulated signal was subtracted from the dataset and only the added sensor noise was present prior to source reconstruction. The 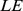 measure was calculated as given by (17).

Reconstructions for a given source location were considered to be significantly better than chance if the signal+noise 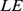 was less than that for the noise-only 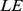 for 95 or more of the 100 added noise samples used in the simulation. On average, 20% of the simulated source locations did not pass the significance test. These ‘non-reconstructable’ source locations were predominantly inside the insular cortex and on the medial walls of both cortical hemispheres, where sources are deep, cortical surfaces touch, and therefore the solution is particularly ambiguous. Cyan-colored spots mark such locations in Figure 3.

#### Amplitude ratio 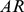


The amplitude ratio measure 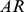 given by (18) quantifies how well the relative source strength between the left and right sources was reconstructed. The true ratio was 2, which is indicated by the dashed line in Figure 4. Mean 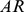 values averaged over all 2-source configurations are shown in Figure 4. Overall, Harmony reconstructed the true amplitude ratio the best among the tested algorithms. MNE had a comparable performance.

#### Surface bias 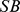


The surface bias measure 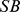 given by (20) is shown in Figure 5. MNE algorithm produced the strongest surface bias (25–30%), while the remaining algorithms produced biases in the range of 10–25%. Differences between the configurations with sources in different hemispheres (shown with solid bars) and those with sources in the same hemisphere (shown with hashed bars) were not very significant. Overall, the same-hemisphere configurations were reconstructed with a slightly stronger surface bias for orientation-constrained sources. Smaller biases were expected for IBF, WMNE, and LORETA, where depth-weighting prior (15) biasing solution toward deeper sources was used. When used without such depth-weighting prior 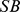 measures for these algorithms were as high as those for MNE (not shown).

More surprisingly, both Harmony algorithms showed relatively small surface bias even though no depth-weighting prior was used in this case. Apparently, Harmony reduces the surface bias by constraining the solution to be smooth - much more so than IBF and LORETA. This forces the solution to extend deep into the sulci. Depth-weighting can increase localization error instead of decreasing it (compare MNE and WMNE results in Figures 2 and 8). This is not surprising given that such a prior indiscriminately biases solution inward even for those locations where it is not justified. Thus, the ability of Harmony to decrease the surface bias without resorting to depth-weighting is noteworthy.

Comparing the middle and right panels in Figure 5 one can see that removing the orientation constraint produced significantly stronger surface bias for all algorithms except MNE. The localization error was somewhat smaller for orientation-unconstrained solutions (Figure 2), hence, although the unconstrained solution was more accurate than the constrained solution it was also more surface biased.

#### Coherence 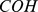


The coherency measure 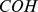 given by (21) was averaged over all 2-source configurations and plotted in Figure 6 for the tested algorithms. Because 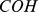 measure is not defined for single-dipole source configuration no corresponding panel is present in Figure 6. The results demonstrate that Harmony in the basis of spherical harmonics (HRM) produced by far the most coherent solutions. Harmony in the basis of spherical splines (SPL) and LORETA follow the lead. The lower coherence of SPL compared to HRM can be explained by the discrete nature of the spherical splines: SPL solutions sometimes produced grainy ‘hotspots’ pulled apart among the neighboring spline centers, as illustrated by Figure 1.

Removing the orientation constraint produced more coherent solutions overall (compare right and left panels in Figure 6). The improvement was most noticeable for the LORETA, MNE, and WMNE algorithms. The unconstrained source reconstructions indeed look smoother and more coherent for these algorithms if the dipole magnitude only is considered. This change is easy to understand: while the orientation constraint forced the magnitude to go through zero every time the dipole directions reversed across the cortex, removing the constraint allows the reversal to happen by mere rotation of the dipoles. The dipole directions still show the same incoherent variation across cortex as for the constrained solution (e.g., across sulci) but this variation is not reflected by the 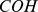 measure which takes into account only the dipole magnitudes.

#### Congruency (width – error correlation) 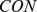


The congruency measure 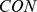 given by (22) was averaged over all 2-source configurations and plotted in Figure 7 for the tested algorithms. The results demonstrate that HRM and MNE solutions had the highest congruency overall, closely followed by SPL. The best congruency measures were close to 80%. This makes Harmony solutions highly indicative of their localization precision. Removing the orientation constraint improved congruency for depth-weighted algorithms (IBF, LORETA, WMNE), but decreased congruency for the rest of the algorithms.

#### Area under the ROC curve 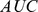


The 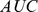 values plotted in Figure 8 give the probabilities of finding higher-amplitude dipoles inside the source patch, quantifying the degree of overlap between the true sources and their reconstructions. As discussed in the Materials and Methods section the measure becomes small and oversensitive (noisy) when applied to small source patches used here (for this reason results for the single-dipole sources were not shown at all). Thus, one can note from Figure 1 where 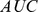 values were plotted next to the reconstructions that the measure changes about two-fold between the HRM and SPL reconstructions even though they appear very similar. Also, comparing Harmony solutions to those by other algorithms one can notice that the 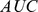 measure does not penalize scattered (incoherent) reconstructions. In fact, it is easy to see from its definition (23) that 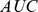 only accounts for the ‘amount’ of the solution that missed the true source not for its separation from the true source. This shows that 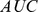 cannot serve as a substitute for the localization error, 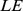, nor for the coherence, 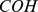.

Comparing 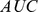 values in Figure 8 one can see that for the orientation-constrained reconstructions HRM and SPL were, overall, on par with LORETA and IBF reconstructions and significantly better than MNE and WMNE reconstructions. For the orientation-unconstrained reconstructions Harmony clearly outperformed other algorithms. 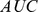 measures were significantly lower for configurations where sources were in the same hemisphere (shown with hashed bars) compared to configurations with sources in different hemispheres (shown with solid bars). This result is not surprising given that sources were overall closer to each other for the same-hemisphere configurations, which resulted in more confounded scalp potentials. Other than the overall magnitude, the pattern of 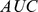 measures was the same in this case: HRM and SPL were on par with LORETA and IBF for orientation-constrained reconstructions and best performers for unconstrained reconstructions.

#### Solution normalization

The MNE solutions have a strong surface bias which can be reduced by depth-weighting (Figure 5). Alternatively, one can reduce the bias by dividing (normalizing) the solution with a measure of the bias for each current dipole. [15] proposed to use the estimated standard deviation of the current (due to the measurement noise) as the measure of the bias. The normalized MNE solution has the meaning of Z-score and was hence termed “dynamic statistical parametric map” (dSPM). Alternatively, [14] proposed to use the square root of the diagonal of the model resolution matrix 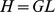 (see Methods for the definitions of 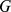 and 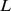) for the MNE normalization measure (sLORETA). This normalization approach was designed to produce zero localization error for a single point source and noiseless measurement.

Because dSPM and sLORETA are popular approaches to source localization a comparison between these methods and Harmony is presented here. To this end, Harmony solutions (spherical harmonics basis and spherical splines basis) were normalized dSPM-style, i.e., by the standard deviation of the solution's noise estimated for each current dipole. Variance of the solution was calculated as given by (13). The results for the 37-patch orientation-constrained condition are shown in Figure 9. The same six measures used to evaluate the quality of the source reconstruction were plotted in the individual panels of the figure in the same order as they were presented in the above sections. The results for the single-dipole and orientation-free conditions showed altogether similar trends and were not displayed.

The localization accuracy (top left panel) was significantly improved by normalization (compare with Figure 2 middle panel). The cumulative 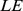 distributions shown in the panel were very similar among the compared methods, however, normalized Harmony solutions denoted as nHRM (spherical harmonics) and nSPL (spherical splines) produced somewhat smaller errors overall than dSPM and sLORETA, especially for the best-localized sources. The small Harmony advantage was more pronounced for the orientation-free condition (not shown), in which case 55% of two-source configurations were localized with better than 3 cm precision for Harmony versus 45% for dSPM/sLORETA. The surface bias (top right panel) was much reduced by the normalization for MNE solutions (dSPM and sLORETA), the reduction was also significant for Harmony solutions. This confirms the efficacy of the normalization technique in this respect for all tested algorithms. On the other hand, the normalization adversely affected congruency which was reduced for Harmony and MNE solutions but significantly more so for the latter. The rest of the measures were only slightly affected by the normalization. Harmony solutions had advantage over MNE solutions in terms of source amplitude ratios (close to 2) and much higher coherence, the AUC measures were about the same for all tested algorithms.

### Real data

The Harmony algorithm was also tested with real EEG data. The EEG recordings were done using HydroCell GSN 128-channel system (EGI Inc.). Visual and tactile sensory stimulation were used in two separate experiments. The stimuli were: a full-screen grating contrast reversing at 1 Hz for the visual experiment and a 2 Hz vibrotactile stimulation of the left index finger for the tactile experiment. ERP epoch duration was 1000 msec and 500 msec respectively. The tactile pressure was applied for the first 100 msec of the epoch and then the pressure was released. 200 stimulation epochs were recorded for each experiment, the epochs were blocked into 10 epoch trials separated by no-stimulus intervals 3 seconds long. Epoch start markers were recorded along with the EEG data using a DIN signal generated by an in-house stimulation software. About 5% of epochs were rejected as too noisy based on potential thresholding at the preprocessing stage. The remaining epochs were averaged. Two different subjects participated in these experiments. Their BEM head models were constructed from high-resolution anatomical MRI scans as described in Materials and Methods. The noise covariance matrix 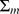 was estimated by projecting out the averaged stimulus epoch from the raw data. The remaining signal was used as a noise estimate to calculate 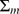. Ordinary cross-validation (OCV) was used to set the regularization parameter for both experiments as described in the Materials and Methods section. The orientation constraint was used for reconstructions.

Experimental data and source reconstructions are shown in Figure 10. The top row shows results for the visual experiment, the bottom row - for the tactile experiment. A snapshot of ERP data is displayed on a flattened scalp surface (the leftmost panels), the colors indicate potential values (hot - positive, cool - negative) with respect to the average reference. ERP time course at the location marked with a white dot is transparently overlaid on the scalp surface. The red arrow indicates the time point at which the scalp snapshot was taken (97 msec for visual stimulation, 86 msec for tactile stimulation, N1 ERP component in both cases). Likely sources of these early ERPs are shown in the next panel to the right. Left and right V1 (primary visual cortex) determined for this subject based on fMRI retinotopy are shown by the green patches overlaid on the inflated cortex (top row). The postcentral gyrus is displayed in cyan, its midsection, where the index finger response is expected to occur in the primary somatosensory cortex [39] is shown in green (bottom row). HRM source reconstruction results are shown next. The SPL solution looked almost identical to the HRM solution. For comparison, MNE results are shown next to the Harmony's. Solutions by the remaining algorithms produced activation patterns qualitatively similar to those by MNE (compared to Harmony solutions) and are not shown here. The Harmony reconstruction of visual activations (top row) closely overlaid the V1 areas as well as the adjacent V2 and V3 areas, which were all likely cortical sources at this response latency. The Harmony reconstruction of the tactile activations were also in fair agreement with the expected location. The same as for the simulated data the MNE solutions appear more incoherent compared to the Harmony solutions, and show the typical sign reversal across sulci.

## Discussion

A new approach to EEG/MEG source reconstruction based on choosing a parsimonious subset of basis functions for the source space was presented. Two basis sets were tested: spherical harmonics and spherical splines. The main advantage of this approach is the explicit way in which the basis set for the solution is chosen. It allows making the solution spatially smooth reducing surface bias and effects of sensor noise. The method's performance was evaluated based on simulated and real EEG data and was compared with performance of several popular source-localization algorithms.

The proposed method, called Harmony, produced realistic source reconstructions when applied to EEG data collected in two different experiments. The reconstructions were based on individual BEM head models derived from anatomical MRI data. The obtained solutions were smooth and coherent, showed little surface bias, and were located over the expected cortical sources (Figure 10).

**Figure 11:**
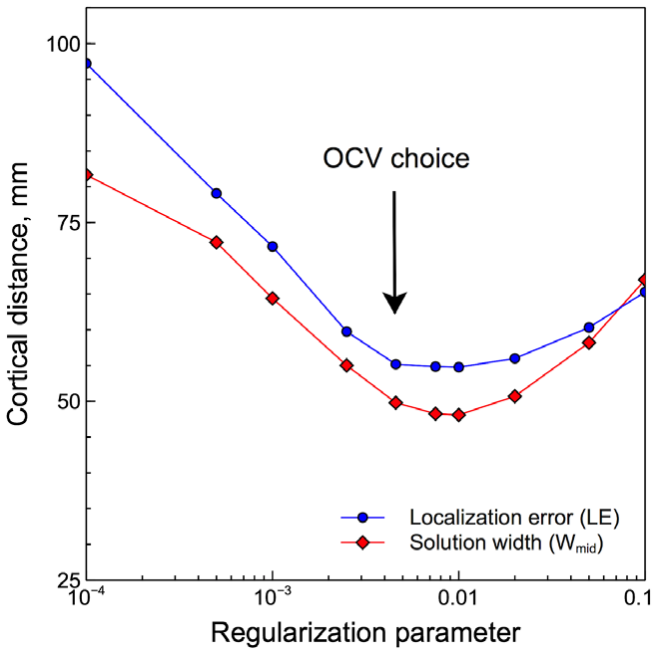
OCV choice of the regularization parameter sets Harmony's (spherical harmonics basis set) mean localization error and solution width close to their minima. The curve was obtained by averaging the measures over all 2-source simulations.

The algorithm was also tested with carefully simulated data. Two simultaneous cortical sources were used in the simulations. The sources had unequal amplitudes and were positioned at 66 uniformly spaced locations each, both on the opposite and on the same cerebral hemispheres. Scalp potentials produced by the two sources were calculated using a BEM head model derived from group-averaged anatomical MRI data provided by FreeSurfer. Noise recorded in a real EEG experiment was added to the simulation and was also used to estimate the statistical significance of solutions. Harmony solutions were compared with solutions for other algorithms: IBF, MNE, WMNE, dSPM, sLORETA, and LORETA. A special emphasis was made on choosing the regularization parameter individually for each of the tested algorithms using OCV. It was a significant factor for the comparison given that the OCV-estimated parameter varied by the factor of 20 among the algorithms. OCV was shown to provide near-optimal regularization in terms of the localization error and the solution width (Figure 11).

The algorithms were compared on the following six measures: localization error (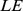), amplitude ratio (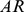), surface bias (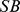), coherence (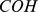), congruency (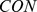, width – error correlation), and area under the ROC curve (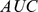). The two Harmony methods (spherical harmonics and spherical splines) provided the best solutions overall. The solutions had the smallest localization error along with the best amplitude ratio, largest coherence and congruency. Harmony algorithms were on par with other algorithms on the 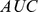 measure for orientation-constrained reconstructions and outperformed the other methods once the constraint was removed (Figure 8). About half of the tested source configurations were localized with better than 4 cm precision (Figure 2).

Harmony solutions had surface bias significantly weaker than that for MNE, about the same as IBF, LORETA, and WMNE solutions (Figure 5). While these algorithms used a depth-weighting prior to decrease surface bias no such weighting was necessary for Harmony. MNE showed second best result on localization error, amplitude ratio, and congruency, but the rest of the scores were modest or poor (e.g., coherence, 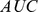). Depth-weighted algorithms (IBF, LORETA, and WMNE) showed relatively poor results overall. In particular, WMNE and IBF were lacking in localization precision and coherence but performed well on the surface bias score. This shows that depth-weighting only guarantees deeper solutions, but not necessarily better reconstructions. IBF and LORETA produced similar results overall, which is not surprising given their similar choice of a smoothness constraint for source reconstruction. The larger localization error for WMNE compared to MNE appears to contradict the results of a study where depth-weighting was shown to decrease the localization error [12]. This difference is likely due to the different definitions of the localization error used. [12] used 3D Euclidean distance between the true source and its reconstruction while the current study used a 2D distance estimated along the surface of the cortex (see Methods). For the fairly common case of the sulcus source mislocalized into a neighboring sulcus depth weighting can make the reconstruction closer to the true source in the Euclidean sense by pushing it deeper into the neighboring sulcus while at the same time separating it farther away along the cortical surface (see Figure 1 in [12] for an illustration).

The algorithms were tested with and without orientation constraint. When the orientation constraint was used, only dipoles orthogonal to the cortical surface were allowed in the solution. Arbitrary dipole orientations were allowed for unconstrained solutions. Although unconstrained solutions somewhat decreased the localization error, increased coherency, and 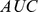 measures, these solutions were also characterized by larger surface biases.

The algorithms were separately tested for source configurations comprising two sources located in opposite cortical hemispheres and in the same hemisphere. Only the 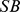 and 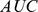 measures were applicable to the latter case. While the surface bias increased only slightly between the opposite-hemisphere and same-hemisphere sources, the overlap of the solution with the true sources described by the 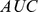 metric dropped very significantly.

MNE solution normalization (dSPM and sLORETA) significantly reduced localization error and surface bias. Adversely, the solutions' congruency was also strongly reduced indicating that the spatial extent of dSPM and sLORETA reconstructions was less indicative of the localization accuracy compared to MNE. This result is in agreement with the increased source dispersion metric between MNE and dSPM/sLORETA [37]. Coherence and source amplitude ratios were largely unaffected by the normalization. dSPM-style normalization had largely the same effect on Harmony solutions except that the reduction in congruency was less pronounced. Overall, the characteristic features of the Harmony reconstructions (coherence, congruency, localization accuracy) characterized the normalized solutions as well.

The high coherence (low scatter) of the Harmony solutions is a unique feature of this approach. Figure 1 demonstrates that for the two simulated sources Harmony algorithms produced two distinct hotspots of activity where the other algorithms produced multiple hotspots. This makes it possible to use the results of Harmony reconstruction as a starting point for ‘parametric’ approaches: several dipoles (or multipoles/patches) can be fitted into the data by first positioning them over the Harmony hotspots and then using an iterative optimization routine to find the best-fitting locations and amplitudes. Harmony and parametric algorithms can be even combined in a recursive manner: Harmony results can be used to seed dipoles for the ‘parametric’ fit and then the results of the fit can be used as sparse priors for the final Harmony reconstruction. Such hybrid approach could get the best of both worlds, e.g., significantly decrease the surface bias while staying within the framework of the ‘distributed’ solutions. This approach is currently being investigated.

Harmony in the basis of spherical harmonics (HRM) produced reconstructions almost identical to those for the basis of spherical splines (SPL), which are in many aspects very different basis sets. This suggests that there is nothing special about a particular choice of the basis set functions as long as the functions share certain generic characteristics, such as smoothness and global extent. HRM significantly surpassed SPL only on the coherency score. HRM was also more parsimonious, it used 121 basis functions per hemisphere compared to 162 basis functions for SPL. Among other things, such a compact description provides an efficient way to store and share source reconstruction results. In addition to the left and right cortical meshes on which any cortex-based source reconstruction is defined, an orientation-constrained HRM solution requires to store only 242 numbers for each time sample. For example, assuming 2-bytes per data record, the whole 4D ‘movie’ of source reconstruction comprising 1 second long ERP epoch sampled at 500 Hz can be saved as a single 522 KB file: 242 KB of HRM solution + 280 KB required to describe 20,000-strong mesh of cortical nodes for a given subject).

Another interesting aspect of the HRM solution is its power spectrum, i.e., the distribution of the source power across different spatial frequencies. Although this aspect was not discussed in this study it provides a new viewpoint on brain activity. For example, it allows to correlate temporal and spatial dynamics of brain rhythms to search for traveling waves of activation [40], [41].

## Materials and Methods

### Ethics Statement

The part of the study which involved human subjects was conducted in accordance with the institutional IRB guidelines. The study was approved by the Northeastern University Institutional Review Board. A written informed consent (approved by the IRB) was obtained from all human subjects.

### Source basis set

The forward EEG/MEG problem for the ‘distributed’ solution is defined by the gain matrix 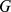:
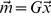
(1)where 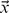 is the vector of the unknown dipole amplitudes (sources), and 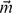 is the vector of sensor measurements for a given time point. The dipole positions on the cortex (and, optionally, their orientations) are fixed. Typically, the dimension 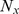 of the source space is two orders of magnitude larger than the dimension 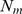 of the sensor space. 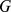 is obtained by solving the Poisson's equation for a given head model, e.g, 4-sphere, BEM, or FEM (finite-element model).

Assuming normal distribution for 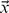 and following the usual Bayesian procedure, the most likely 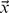 fitting the data 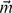 is given by the following inverse solution [42]:
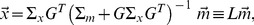
(2)where 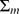 is the measurement noise covariance matrix (assumed to be known from the data), 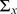 is the (unknown) source covariance matrix, and 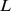 is the inverse solution matrix. The source covariance matrix defines the prior for the source distribution and hence its choice can strongly affect the solution. Indeed, the choice of the source priors is the true “hard” problem of source localization. To begin with, the degree of the solution regularization is determined by the overall scaling factor of the source covariance matrix. Otherwise, the elements of 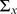 define the constraints which are applied to the underdetermined inverse problem. The various ‘distributed’ source localization algorithms mentioned in the Introduction differ mainly in their choice of the source covariance matrix. If 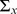 is taken to be an identity matrix, one obtains the minimum-norm solution. By choosing a different source covariance matrix one deviates from the minimum-norm constraint. For example, one may introduce higher variances for deeper sources, which results in biasing the solution to those deeper sources. However, this does not guarantee that the solution will be closer to the true sources, it only guarantees that the solution will have less of the surface bias typical for the minimum-norm solution. One recent approach is to learn the elements of 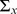 from the data [23], [43]. While this approach significantly improves on the results of the earlier algorithms (WMNE, LORETA), it is not guaranteed that the data *per se* provides all the necessary constraints to make the solution plausible.

The approach presented here seeks the solution in the form of an expansion into a small set of global continuous and smooth basis functions instead of starting from the ten-thousand-strong set of discrete cortical dipoles. Because of the well-known lowpass spatial effect of the skull on electric currents high spatial frequency components of cortical sources simply cannot be reliably inferred based on scalp potentials due to the potentials being swamped by high spatial frequency sensor noise. Arguably, the proposed choice of basis functions forces the solution to better represent the available information about brain activity and reduces effects of sensor noise.

Consider a linear transformation 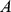 defining a new basis set in the source space and mapping it to the basis set of the dipole amplitudes:
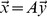
(3)The gain matrix in the new basis set is given by
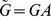
(4)If the basis vectors of the new basis set are global, i.e., they are given by a linear combination of thousands of dipole amplitudes located all over cortex, and if the cortical sources are globally independent, 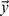 is normally distributed irrespective of the actual statistics of the local cortical currents (the Central Limit Theorem). Note that although the normality assumption underlies the dipole-basis solution (2) its validity for 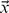 is rather questionable, because individual neurons are non-Gaussian and nearby neurons are usually strongly correlated, which makes their local ensembles 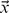 non-Gaussian as well.

Writing the solution (2) for the new basis set one obtains
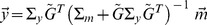
(5)Substituting (3) and (4) in the above formula maps the solution 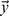 back to the dipole basis:
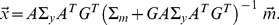
(6)The solution is defined in terms of the source covariance matrix 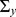 in the new basis set now. Comparing (2) and (5), one can see that the corresponding source covariance matrix in the dipole basis set is given by
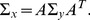
(7)By choosing different 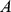 and 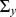 one obtains different solutions to the source localization problem. This demonstrates that different constraints embodied in the choice of the source prior 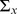 can be viewed as different choices of the source space basis set 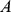. For example, the minimum norm solution is obtained for any basis set 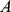 of dimension 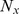, which is complete and orthonormal, if 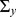 is equal to 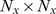 identity matrix 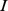. In this case
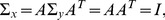
(8)an (6) reduces to the minimum norm solution
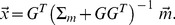
(9)This result applies to the basis set of individual cortical dipoles as well as to a basis set of global orthonormal functions, such as spherical harmonics.

### Choice of the source basis set

Because EEG/MEG sources are spread on topologically spherical cortices (ignoring the corpus callosum) it is natural to use spherical harmonics or spherical splines as the basis set functions, the method termed Harmony here. Between the two basis sets spherical harmonics produced somewhat better results, but the differences were small.

Spherical harmonics are 2D analogs of the sine and cosine functions, and the spherical harmonics expansion is a 2D analog of the conventional Fourier series expansion. The spherical harmonics define a complete orthonormal set, each vector is global (spatially extended) and is characterized by a spatial scale determined by the Harmonic's 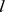 index. The index defines the number of node lines between South and North poles. The 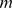 index runs from 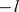 to 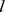 and determines the number of node lines along the Equator. The total number of spherical harmonics (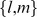 pairs) for a given 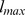 is 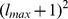. Higher indices correspond to higher spatial frequencies. The corresponding transformation matrix 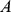 is given by
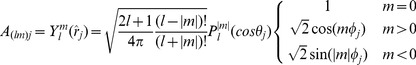
(10)Here the row index 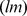 enumerates spherical harmonics (all combinations of 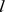 and 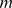 indices), while the column index 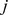 indexes the dipole locations on the cortical surface, 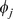 and 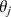 are the azimuth and elevation of the 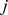-th source dipole on a cortex inflated into a sphere. 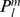 are the associated Legendre polynomials.

**Figure 12:**
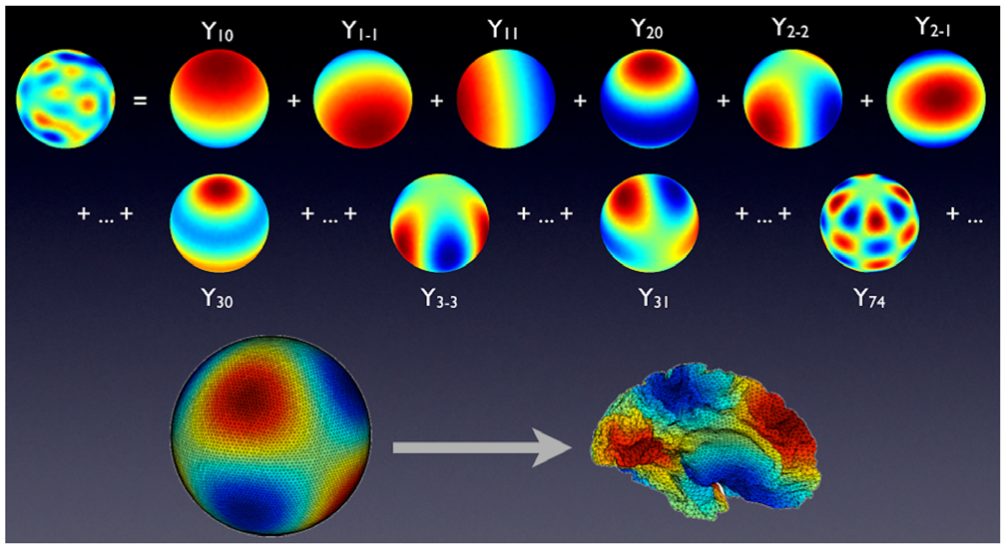
Top: Expansion of a given source distribution into a set of spherical harmonics. Bottom: left cortex displayed as a spherical mesh with a spherical harmonic computed at its nodes, then the mesh is folded into the actual cortical shape (as produced by the FreeSurfer toolbox). The colormap indicates the source sign and amplitude.

Any square-integrable scalar function defined on a spherical mesh can be expanded into a set of spherical harmonics, as is illustrated in the top of Figure 12. Given that each cortex is topologically a sphere (folded into the pial shape), one can use the spherical harmonics basis to span the source space for each cortex. Hence the full 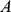 matrix is block-diagonal, its two blocks corresponding to the spherical harmonics applied to the left and right cortices respectively. The gain matrix 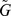 is calculated based on the dipole locations on the actual folded cortices, of course. This is illustrated in the bottom of Figure 12.

Some degree of non-orthonormality will be introduced into the basis set by the folding distortions of the spherical mesh, but this is not a concern because strict orthonormality is not a requirement for a source basis set, although non-orthonormality can potentially affect the stability of the numeric solution of (6). Moreover, the fact that the basis set of spherical harmonics produced solutions almost identical to those obtained in the basis set of spherical splines (11), which was not at all orthonormal and otherwise very different from the basis set of spherical harmonics, indicates that variations in the basis functions due to the folding mesh distortions were probably inconsequential.

Because EEG/MEG sensors sample signals at discrete spatial locations (typically, about 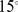 apart), the basis set of spherical harmonics required to describe scalp potentials (measurement space) has a natural cutoff frequency defined by the corresponding Nyquist limit. For the typical high-density EEG cap of 128 electrodes this frequency corresponds to 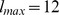 or 169 harmonics altogether. If cortex was modeled as a sphere one could simply use the same cutoff index for the basis set of spherical harmonics on cortex (source space). However, in the described approach the spherical harmonics were applied to two folded spheres (the left and right cortices) and the cutoff index had to be found empirically. Because increasing 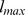 above 10 did not significantly reduce the source localization error (17) in the simulations, 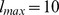 was used as the cutoff index for each cortex. This gave the total of 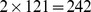 harmonics. Note that increasing the density of sensors does not necessarily increase the cutoff index. The low-pass spatial filtering effect of the skull suppresses high spatial frequency signals and makes the neighboring sensors strongly correlated. Besides, because of the low-pass filtering of the skull the high-frequency harmonics quickly become dominated by the sensor noise and therefore the input of these harmonics into the solution should be suppressed.

The proposed choice of the source space basis is by no means unique. The basis of spherical harmonics allows to fit any measured data without introducing high-frequency information not present in the data as defined by the Nyquist limit. Arguably, any other choice of a basis set with similar properties would result in the same or very similar inverse solution. Indeed, the simulation results presented here indicate that the basis set of spherical splines produced a solution very similar to the basis set of spherical harmonics, even though the basis functions of the two sets were very different: spherical harmonics are periodic, while spherical splines are centered.

The spherical splines 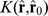 were constructed using the Abel-Poisson kernel, which has the following closed form expression [44]:
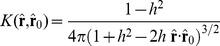
(11)where 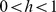 is the spline scale parameter (analogous to 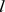 for spherical harmonics), and 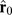 and 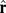 stand, respectively, for the spline center position and the point in space where the spline needs to be calculated.

### The choice of 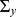


The 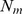 vectors spanning the solution subspace of the 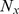-dimensional source space are given by the columns of the numerator matrix in (6). If the covariance matrix 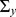 is proportional to the identity matrix (i.e., the priors for all spherical harmonics are equal), the numerator is just a projection defined by 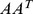 from the minimum-norm subspace spanned by the rows of the gain matrix 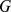 to the subspace defined by the spherical harmonics basis. Although this simple choice of the 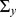 prior works well for localizing noiseless data, significant amounts of sensor noise can result in spurious sources. Harmony provides a simple solution to the noise problem. Because the sensor noise is usually very local it appears mainly at high spatial frequencies. The noisy high frequency signal can be progressively ignored by the solution via scaling down the diagonal elements of 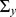 for high-frequency harmonics, for example as 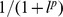. The exponent 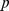 can be left as a free parameter and normally falls within the 0.5–1 range, depending on how noisy the data is.

Although the described choice of 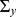 is *ad hoc* its effects are well-controlled. The 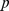 parameter simply biases the solution to lower spatial frequencies. Ideally, one would like to set the diagonal values of 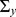 more objectively, for example by learning them from data as the model hyperpriors [43]. To this end the covariance matrix 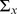 can be written as
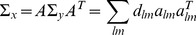
(12)Where 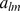 are the columns of the 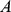 matrix and 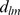 are the diagonal elements of the (diagonal) 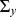 matrix. In this form 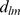 appear as the model hyperpriors and can be learned by maximizing the probability of the observed data with respect to the hyperpriors. For example, in the current scheme the single hyperprior 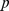 can be learned this way. The results of this approach are not discussed here.

### Head model

BEM head models were constructed based on high-resolution MRI data collected for two subjects and also using the FreeSurfer group averaged head (MRI data averaged over 40 subjects). The BEM head model comprised three volumes: scalp, skull, and CSF/brain; the volumes were reconstructed with the help of the FSL toolbox [45]. The corresponding BEM gain matrix 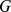 was calculated using the MNE suite toolbox [6]. To improve the BEM precision the inner skull was meshed as the 5-th subdivision of icosahedron (20,480 triangles), the outer skull and scalp surfaces were meshed as the 4-th subdivision of icosahedron (5,120 triangles). Relative conductivities of the three volumes were set to (1, 1/30, 1) for scalp, skull and CSF/brain respectively.

Cortical surfaces and ROIs were determined by automatic segmentation with the help of the FreeSurfer toolbox [46] and fMRI-based retinotopy [47] with the help of the FSL toolbox [45]) respectively. In particular, the FreeSurfer toolbox was used to obtain pial, mid-gray, inflated, and spherical (fully inflated) cortical representations. The FreeSurfer spherical meshes were used to define the 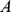 and 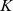 matrices given by (10) and (11) respectively. 10,242 current dipoles were positioned at the nodes of a triangular mesh (5-th subdivision of icosahedron, mid-gray FreeSurfer mesh) for each cortex. The localization results are shown on slightly inflated cortices in all figures. The amplitude and direction of the cortical currents are represented by color intensity and hue: inward (cold) and outward (hot). A colormap similar to the one used by the FreeSurfer and MNE toolboxes was used.

### Source basis set

Two basis sets: spherical harmonics and spherical splines were used with the Harmony method. The spherical harmonics basis set was defined by (10). 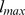 was set to 10, which created a basis set of 121 harmonics on each cortical surface. Larger 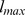 values did not significantly decrease localization errors. 162 spherical splines defined by (11) were uniformly positioned on each cortical surface with 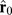 located at the nodes of the second subdivision of icosahedron meshing the spherical cortical surface.

### Sensors

A special care was taken to make the simulations as realistic as possible. Electrode locations were taken from a real EEG experiment, in which a 128-channel HydroCell GSN electrode net was used (EGI Inc.). The electrode positions were measured using a Polhemus FASTRACK digitizer.

### Sources

Because, typically, several cortical sources are simultaneously activated, two simultaneously activated sources were used in the main bulk of simulations. The sources located in opposite cerebral hemispheres and in the same hemisphere were analysed separately. Two source configurations were tested: point-like and extended. For the point-like configuration, each of the two sources consisted of a single current dipole. Although this configuration is widely used for simulations, it is not very realistic. The neurologist's “rule of thumb” is that at least 6 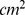 of cortex has to be active to record scalp potentials without averaging [1, ch. 1.8 and references therein]. Correspondingly, for the extended configuration each of the two sources included a single dipole and all its nearest neighbors up to the third-degree coordination number, which gave 37 dipoles altogether: 1+6+12+18 = 37. The patches were roughly hexagonal in shape approximately 2.5 cm in diameter when measured along the cortical surface. This corresponds to 5 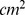 of cortical area, close to the “rule of thumb” size.

The dipole orientations were fixed to be orthogonal to the cortical surface, which reflects the common assumption that EEG and MEG are due to synaptic currents produced by activity of cortical pyramidal cells. These currents flow along the cells axons primarily perpendicular to the cortex.

The single-dipole and hexagonal 37-dipole patches are illustrated by insets in Figures 2–8. A pair of the extended patches is visible in Figure 1, where each patch shown as a green shape is overlaid on inflated cortical hemispheres. 66 uniformly spaced patch locations were chosen for each cortex, separation between the nearest-neighbor patch locations were close to the patch diameter. This defined 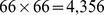 dual-source configurations altogether. One source always was half the amplitude of the other source, which simulated source amplitude variation in the actual brain. The direction of the source currents was outward with respect to the cortical surface for both sources. When the two patches were in the same hemisphere the configurations where they fell into the same locations were omitted from the simulations. In this case 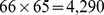 dual-source configurations were simulated for each hemisphere. The electric potentials on the 128 scalp electrodes were obtained using gain matrix 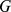 calculated based on the FreeSurfer group-average BEM head model. The strength of the source dipoles was chosen such as to produce the scalp potentials of the order of 10 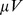, which is the typical order of magnitude for evoked potentials.

The reconstructed sources were thresholded based on the signal-to-noise power ratio (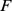-statistics). Only the sources passing the null-hypothesis test (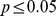, Bonferroni corrected using the number of sensors, 128 here [16]) were colored in Figures 1 and 10. The 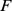-measure was calculated as the ratio of the squared cortical current 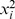 to the estimated variance of the solution's noise 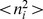 for a given dipole location 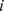. The solution noise variance was calculated by linearly propagating the measurement noise covariance matrix 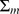 onto the cortex:
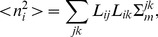
(13)where 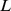 is the inverse solution matrix given by (2), 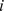 numerates the rows of the solution matrix and 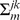 stands for the 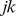 element of the measurement noise covariance matrix. Because the number of degrees of freedom for the 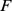
's denominator (determined by the number of observations) is much larger than that for the numerator (1 for an orientation constrained solution, or 3 for an orientation-free solution), the 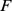 statistics can be safely approximated by a gamma-distribution statistics, which only depends on the degrees of freedom of the numerator.

### Noise

Real experimental noise was added to the simulated sensor potentials. The noise was recorded in the course of a VEP (visually evoked potentials) experiment, where a contrast reversing checkerboard was occasionally replaced by empty gray background with a fixation mark. Subjects were instructed to fixate at the mark and avoid any head-muscle or eye-muscle activity during the trials. Each trial lasted for 10 seconds and comprised ten 1000 msec VEP epochs (typical epoch duration for an evoked response experiment). The whole experiment lasted for 40 minutes. The “empty screen” epochs (190 altogether) were averaged to obtain the estimate of the residual noise in the averaged VEP epoch. The noise amplitude was in the 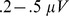 range and did not exceed 1 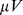. 100 randomly chosen samples of noise were added to the simulated sensor data for each source configuration and the resulting solutions averaged to estimate the solution for noisy data.

### Regularization

Because the source covariance matrix 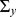 has the meaning of a prior for the source variance [42] it can only be defined up to an unknown scaling factor. Given the position of 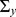 in (5), mathematically, the scaling factor has the meaning of the Tikhonov regularization parameter [18]. Hence the scaling factor determines the balance between fitting the observed data and fitting the Gaussian prior, which has its most likely value at zero, i.e., no sources.

In practical terms, weak regularization results in unrealistically patchy high-amplitude solutions which, nevertheless, fit the data very well. Strong regularization results in diffuse and low-amplitude solutions which produce scalp potentials lower than the ones actually observed. As mentioned in Section “The choice of 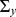
”, Harmony allows to reduce effects of noise by scaling down the diagonal elements of 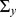 as a function of the spherical harmonic index 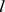 via the scaling parameter 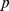. The scale parameter 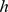 in (11) has the same effect on the solution in the basis of spherical splines. 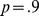 and 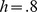 were found to minimize the localization error of Harmony solutions and these values of the smoothing parameters were used for our simulations. The overall scaling (regularization) factor for 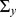 needs to be determined for Harmony as well as for other algorithms, but thanks to the smoothing parameters 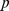 and 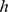 effects of regularization on the solution become more controllable.

It is important for the purpose of comparison of different source localization algorithms to choose the regularization parameter intelligently. Simply setting the regularization parameter to the same value for all algorithms would not do because it would affect different algorithms differently. Hence the parameter has to be set by some optimization scheme applied to each algorithm individually. There are many such schemes available [18], [48]. Ordinary Cross Validation, OCV, [49] was chosen for this purpose in the present study. In the author's experience, OCV works better than its more popular approximation, General Cross Validation (GCV) because OCV rarely produces local minima in the cost function, which are common for GCV. The regularization parameter determined by OCV produced reasonable-looking solutions when applied to real EEG data. As Figure 11 demonstrates, regularization chosen by OCV also nearly minimizes measures of the solution's localization error 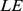 and width 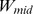 for the simulated data. These measures are defined in the following sections; the effect of OCV on the other measures was not investigated.

The OCV method uses “leaving-out-one” validation strategy, where one datapoint at a time is left out, the remaining data is fit by the model, and the misfit of the left-out datapoints is minimized. The procedure can be reduced to a single formula: the optimal regularization parameter is given by minimizing the OCV cost function
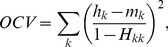
(14)where the summation runs over all datapoints, and 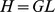 is the 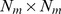 resolution matrix. The numerator of the above formula is a quadratic measure of misfit of the observed data 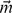 by the fitted model 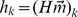. The numerator penalizes data misfit while the denominator penalizes data overfitting (perfect fit corresponds to 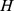 equal to an identity matrix).

### Quantitative comparison with other algorithms

Harmony performance for simulated data was compared with the performance of the following algorithms: MNE, WMNE, dSPM, sLORETA, LORETA, and IBF. The latter method (Informed Basis Functions) claims the most informative source space basis set, i.e., the set preserving most information about the known source constraints (coherence or smoothness in our case). A Gaussian coherence matrix with 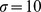 mm was used; the solution on each cortical hemisphere was spanned by the set of 512 IBF basis functions. These parameters were close to the ones used in [19]. Because the solution was constrained to the cortical surfaces for all the tested algorithms a surface (2D) version of the discrete Laplacian was used in LORETA.

WMNE, LORETA, and IBF algorithms employ “depth weighting”. The weighting factor for the 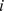-th cortical dipole is given by the following formula:
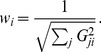
(15)The summation runs over the 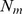 rows of the gain matrix 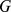. Hence deeper (less effective) dipoles receive a higher prior variance.

Because the main focus of this study was on the choice of the source space for linear algorithms based on the ‘distributed’ solution (2) no comparison was made to algorithms involving various other approaches, e.g., beamformer methods or iterative prior learning methods, such as FOCUSS or MSP. Although these approaches appear promising, they address different aspects of source localization and therefore will not be discussed here. Nevertheless, it is worth mentioning that these new aspects of source localization can be easily combined with the Harmony method.

Six measures were used to quantify the quality of source reconstructions: localization error, amplitude ratio, surface bias, coherence, congruency (width – error correlation), and area under the ROC curve. These measures are explained next.

#### Localization error

The localization error was calculated as follows. First, the location of the true source was calculated by averaging the locations of its 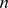 constituent dipoles. 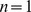 for a point-like source and 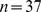 for an extended source. Then, 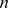 highest-amplitude dipoles were found in the solution for the same cortical hemisphere. For each of the dipoles the distance between the dipole and the source location was calculated along the (pial) cortical surface. To this end the size of the great arc connecting the two locations on the spherical cortex was first found and then converted to the pial cortex distance in mm, 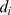. The raw localization error was then taken as the weighted average of 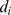:
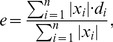
(16)where 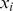 stands for the solution magnitude for the 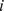-th dipole. Finally, the raw localization error was corrected for the extent of the true source 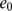, given by the above formula applied to the source patch itself, and averaged between the left and right sources:
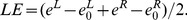
(17)


#### Amplitude ratio

The amplitude ratio measure quantifies how well the relative source strengths are preserved in the solution. The measure was calculated as follows:
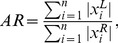
(18)where 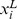 and 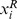 stand for the 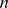 highest-amplitude values in the left- and right- hemisphere solutions respectively.

#### Surface bias

This measure quantifies the degree of surface bias in the solution. First, the solution's distance 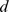 from the “head center” 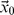 was determined using the following formula
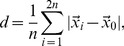
(19)where 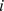 runs over 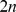 most active dipoles across hemispheres. Then, the relative increase of 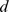 with respect to this distance for the true source, 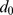, was calculated:
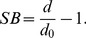
(20)

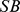
 is zero when the reconstructed source location is at the same distance from the “head center” as the true source location. Positive values indicate surface bias. The “head center” was defined by fitting an ellipsoid into the EEG sensor locations used; the center of the ellipsoid was taken as the head center 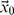.

#### Coherence and congruency (width – error correlation)

The last two measures quantify two important properties characterizing the spatial extent of a solution: its coherence (how ‘scattered’ is the solution) and its congruency. The latter is defined as the correlation between the solution's width (defined below) and its localization error 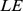. Ideally, the spatial distribution of a solution should be indicative of its precision. Sparse, sharp looking solutions are misleading, if the true source is far away. Therefore, one wants the solution width to be positively correlated with its localization error, and the stronger this correlation - the better.

The solution width is defined first. Its measure is calculated the same way as the localization error, except that the distances 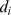 in (17) are measured with respect to the average 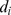 for 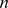 highest-amplitude solution values, i.e., with respect to the center of the solution. Two width measures: 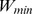 and 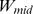 are defined. For the ‘min’ measure the 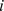 index runs over the strongest 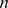 dipoles only. For the ‘mid’ measure 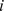 runs over all the dipoles with amplitudes higher than half the maximum amplitude of the solution. Defined this way, 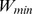 characterizes the width of the solution's ‘hotspot’, while 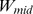 characterizes the solution's spread, which, as discussed above should, ideally, be indicative of the solution's uncertainty. The coherence is defined as
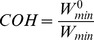
(21)and the congruence as
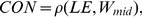
(22)where 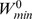 denotes the ‘min’ width measure calculated for the actual source, and 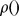 stands for the Pearson's correlation coefficient calculated over all the source configurations used in the simulation. 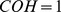 corresponds to the solution having the same ‘hotspot’ size as the size of the simulated source, smaller values indicate a scattered, incoherent solution. The same as for the previous measures 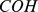 and 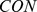 were averaged between the left and right hemispheres.

#### Area under the receiver operating characteristic (ROC) curve

Area under the ROC curve (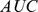) is a popular measure used to evaluate the performance of a binary (hit – miss) classifier. When applied to source localization results, e.g., [36], [50] 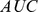 characterizes the overlap between the solution and the true source by measuring the probability of a randomly chosen source vertex (a vertex belonging to the source patch or patches) having a larger activation than a randomly chosen non-source vertex. The 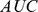 measure can be conveniently calculated as follows [51]:
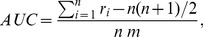
(23)where 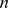 and 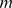, respectively, stand for the number of source patch vertices and ‘non-source’ vertices used for the comparison, and 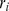 stands for the rank of the 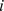-th source vertex. The rank is determined by sorting the source and non-source vertices based on the absolute values of their activations 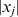, from low to high. It is easy to see that when the values are larger everywhere on the source patch than on the outside 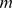 vertices then 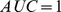. Conversely, if the values are smaller everywhere on the source patch then 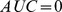. 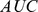 strongly depends on many factors including the source patch size, the number 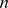 of source patch vertices, the number 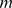 of the non-source vertices chosen for the comparison, as well as their particular choice. Clearly, this choice makes a big difference given that usually there are many thousands of non-source vertices vs. only a handful of source vertices. Typically, the same number of non-source and source vertices is used. The choice of non-source vertices is usually biased toward the most active ones, but the particular scheme varies between different studies. Here 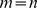 most active non-source vertices were used. Compared to large source patches small source patches defined on dense cortical meshes used in this study naturally have less overlap with a distributed solution and 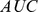 can be quite small in this case. For example, for the case of a single-dipole source 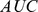 is zero unless the maximum activity vertex in the solution coincides with the source vertex. Obviously, this rarely happens for high-density cortical meshes. For this reason the 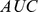 measure was not applied to single-dipole simulations in this study. For the 37-dipole patches both patches were included in the 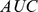 analysis, i.e., 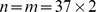. The non-source vertices where always chosen on the same cortical hemisphere as the corresponding source patch. Note that because 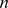 most active non-source vertices were used here the computed AUC values were lower than in those studies where the choice of non-source vertices was less restrictive ([36], [50], for example).
